# Nonmuscle myosin-2: mix and match

**DOI:** 10.1007/s00018-012-1002-9

**Published:** 2012-05-08

**Authors:** Sarah M. Heissler, Dietmar J. Manstein

**Affiliations:** Institute for Biophysical Chemistry, Hannover Medical School, Carl-Neuberg-Str. 1, 30625 Hannover, Germany

**Keywords:** Nonmuscle myosin-2, Regulation, Cytoskeleton, Review

## Abstract

Members of the nonmuscle myosin-2 (NM-2) family of actin-based molecular motors catalyze the conversion of chemical energy into directed movement and force thereby acting as central regulatory components of the eukaryotic cytoskeleton. By cyclically interacting with adenosine triphosphate and F-actin, NM-2 isoforms promote cytoskeletal force generation in established cellular processes like cell migration, shape changes, adhesion dynamics, endo- and exo-cytosis, and cytokinesis. Novel functions of the NM-2 family members in autophagy and viral infection are emerging, making NM-2 isoforms regulators of nearly all cellular processes that require the spatiotemporal organization of cytoskeletal scaffolding. Here, we assess current views about the role of NM-2 isoforms in these activities including the tight regulation of NM-2 assembly and activation through phosphorylation and how NM-2-mediated changes in cytoskeletal dynamics and mechanics affect cell physiological functions in health and disease.

## Introduction

Nonmuscle myosins constitute one of the most abundant and versatile group of molecular motors in eukaryotic cells. Their name is a misnomer as NM-2 isoforms are also present in cardiac, skeletal, and smooth muscle cells, though in much smaller quantities than the sarcomeric myosins. Both during embryonic development and in mature multicellular organisms, NM-2 isoforms act as important regulators of the highly flexible and adaptable actin cytoskeleton [1, 2]. In response to extra- and intracellular cues, the motor activity of NM-2 isoforms contributes to the spatiotemporal organization of the local actomyosin network resulting in contractility and patterning. NM-2 isoforms contribute thus in a critical way to the cell’s ability to respond to changing requirements in order to carry out physiological functions [3].

NM-2 isoforms are conventional members of the myosin superfamily of actin-based molecular motors, one of the largest and most diverse protein families in eukaryotes. The members of the myosin family have been assigned to 35 classes, 12 of them are produced in humans [4]. Conventional or class-2 myosins comprising the so-called skeletal, smooth, cardiac, and nonmuscle isoforms form the largest subfamily [4].

**Fig. 1:**
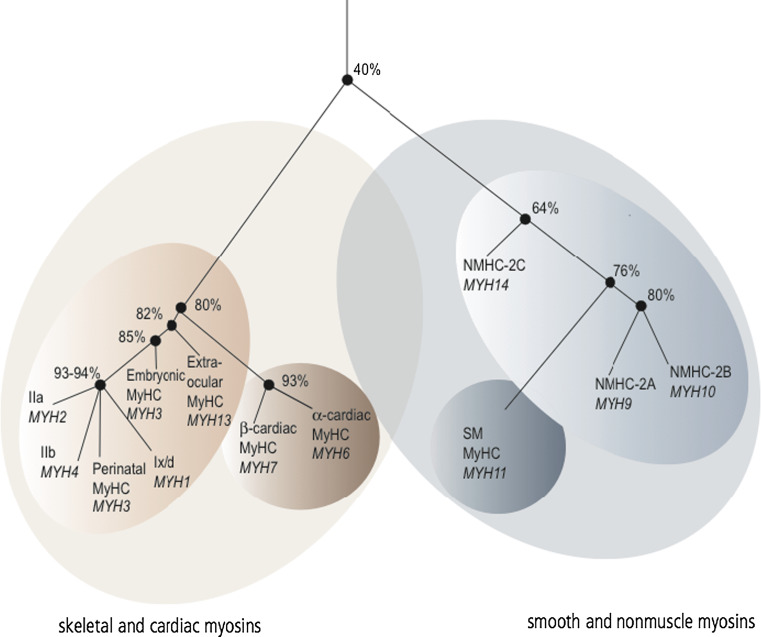
Phylogenetic tree of the myosin-2 subfamily in humans according to Golomb et al. [5]. The numbers adjacent to the nodes define the overall identity of the myosin heavy chains at amino acid level. The myosin-2 family is grouped into skeletal and cardiac myosins as well as smooth and nonmuscle myosins. NM-2C constitutes a distinct branch in latter group and shows an evolutionary closer relationship to smooth muscle myosin than its isoforms NM-2A and NM-2B

NM-2 is a collective term defining three distinct isoforms in vertebrates; nonmuscle myosin-2A (NM-2A), -2B (NM-2B), and -2C (NM-2C). The corresponding heavy chains (NMHC) are encoded by different genes (*MYH9*, *MYH10*, *MYH14*), which are located on three different chromosomes [5–8]. NM-2 isoforms exhibit 60–80 % sequence identity at the amino acid level. Phylogenetic analysis indicates a closer relationship between NM-2C and smooth muscle myosin than between NM-2C and the other NM-2 isoforms (Fig. 1) [5]. Despite a high level of sequential and structural conservation, NM-2 isoforms comprise distinct enzymatic properties and subcellular localizations, suggesting that the isoforms serve specialized cellular functions, even though some cellular functions are interchangeable [9–12].

As downstream effectors of numerous signaling pathways, NM-2 isoform activity and assembly state are tightly regulated. Aberrant regulation and functional impairment of NM-2 isoforms has been associated with the onset and progression of malignancies, including cancer and altered immune response. The prominent role of NM-2 aberrations in disease processes emphasizes the protein’s role in maintaining mammalian homeostasis [13].

Current knowledge about the function of conventional myosins in nonmuscle cells is in part derived from model organisms such as *Dictyostelium discoideum*, *Caenorhabditis elegans*, *Drosophila melanogaster*, and *Xenopus laevis*. *Dictyostelium* and *Drosophila* express a single NM-2 gene, *Dd* *mhcA* and *Dm* *zipper*, making them well suited for genetic analysis and biochemical studies on gene expression, function, and regulation [14, 15]. However, the production of three NM-2 isoforms in vertebrates creates the need for studying NM-2 function and regulation in more complex systems such as mouse models. As outlined below, ablation of NM-2 isoforms in murine models provides the opportunity to study NM-2 isoforms in a tissue-specific and developmentally dependent context and serves as a model system for NM-2-related diseases.

## Structure

**Fig. 2:**
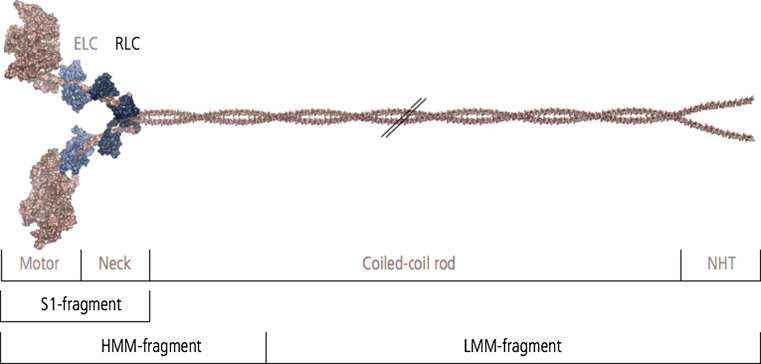
Domain structure and composition of the NM-2 holoenzyme. The NMHC (230 kDa) consists of a N-terminal motor domain, a neck domain, and coiled–coiled domain that terminates in a nonhelical tailpiece (*NHT*). Two NMHC form a homodimer mediated by the formation of the coiled-coil domain within the tail fragments. The enzymatically active motor domain harbors the ATP binding site and the F-actin binding region. The neck domain of each NMHC binds two sets of light chains, the essential (*ELC*, 17 kDa) and the regulatory (*RLC*, 20 kDa) light chain. Tryptic cleavage fragments the NM-2 holoenzyme into the single-headed subfragment-1 (*S1*), double-headed heavy meromyosin (*HMM*) and light meromyosin (*LMM*)

NM-2 is a hetero-multimeric protein complex consisting of a NMHC homodimer that is non-covalently associated with two sets of myosin light chains. At the amino acid level, the NMHC is structurally and functionally characterized by an asymmetric modular organization, containing a N-terminal motor domain, an intermediate neck domain and a C-terminal tail domain (Fig. 2) [16].

The catalytic motor domain harbors the nucleotide binding site as well as the actin binding region and couples the allosteric interplay between ATP hydrolysis and actin interaction, leading to unidirectional movement to the plus-end of F-actin. Vertebrate cells increase the diversity and complexity of the NM-2 proteome by alternative splicing of single pre-mRNA transcripts of *MYH10* and *MYH14* [5, 17]. Splice variants are produced by introducing one or two alternative exons in the mRNA region encoding the myosin motor domain. One alternative exon called B1 or C1, in NM-2B and -2C, respectively, is spliced into the 25- to 50-kDa junction (loop-1) near the nucleotide-binding site [5, 17]. A second exon cassette called B2 or C2 expands the 50- to 20-kDa junction (loop-2) within the actin binding region [17, 18]. All combinations of alternatively spliced exons are possible [5, 17, 19]. The sequential alterations of the myosin motor domain result in the production of kinetically and functionally distinct splice variants in a tissue-specific and developmentally dependent manner.

The neck domain contains two conserved IQ-motifs, which bind accessory light chains. The first IQ motif is occupied by the essential light chain (ELC), and the second by the regulatory light chain (RLC). The ELC stabilizes the NMHC, whereas the RLC has stabilizing and modulating functions. Alternatively spliced ELCs and RLCs were identified, but it is currently not known if there is any isoform-specificity to a given NMHC. Myosin light chains, especially the RLC, constitute an attractive tool to track NM-2 in cell biological studies. The ELC binds to various non-myosin proteins as well as different classes of myosin heavy chains (-2, -5, -6, and -7), whereas the RLC binds to the myosin heavy chains of classes -2 and -18 [20, 21]. This makes the use of antibodies against the RLC to track NM-2 in immunofluorescence studies questionable in tissues that produce myosin-18 along with NM-2. Moreover, Kondo et al. [22] have shown that di-phosphorylated RLC localizes independently from NM-2 and mono-phosphorylated RLC to the midzone during cytokinesis, raising the possibility that tracking of NM-2 via its RLC is misleading.

The tail domain consists of an alpha-helical coiled-coil motif, which terminates in a short nonhelical tailpiece (NHT). The coiled-coil region provides the structural basis for the homodimerization of two NMHC leading to the formation of a rod-like structure. NM-2 homodimers assemble into higher order filaments by patterns of alternating charge distributed along the coiled-coil [23]. NM-2 generally functions as a part of minifilamentous structures, comprising ~28 molecules [24, 25]. By comparison, thick filaments of smooth and skeletal muscle are up to 30-fold bigger. Different from skeletal muscle myosin, NM-2 undergoes dynamic filament assembly/disassembly transitions. The equilibrium is modulated through phosphorylation events, as outlined below. Besides phosphorylation, F-actin appears to directly promote NM-2 filament assembly. Accelerated filament nucleation in the presence of F-actin has been observed for the related *Dd* NM-2, suggesting the spontaneous formation of actomyosin contractile fibers via myosin assembly [26]. Bipolar arrays of NM-2 show directed and processive movement along F-actin, pulling actin filaments of opposing polarity against each other, thereby generating local contractile forces and promoting actin-crosslinking.

It is not fully investigated if NM-2 isoforms form heterotypic filaments. However, the intermolecular assembly of NM-2A and NM-2B rod fragments suggests the formation of heterotypic filaments in vitro [27, 28]. In support, fluorescence spectroscopic studies demonstrate a dynamic exchange of rod fragments between preformed NM-2 homo-assemblies in an isoform-independent manner [28]. Studies from Beach and Egelhoff [29] report NM-2A and NM-2B heterotypic filaments at the contractile rings of dividing cells even though homotypic filaments might be the predominant pool in live cells [30, 31].

## Regulation

**Fig. 3:**
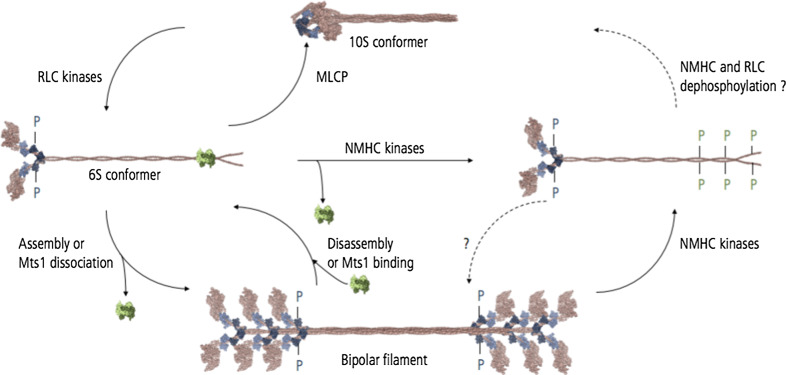
Regulation of mammalian NM-2 enzymatic activity and assembly state. RLC kinases promote the conformational change of the inhibited (*10S*) to the extended NM-2 conformation (*6S*). The inhibited conformation is assembly-incompetent, the extended conformer assembly-competent. MLCP activity shifts the equilibrium towards the inhibited conformation. The transition to the extended conformation triggers the activation of NM-2 ATPase activity and promotes the assembly of NM-2 homodimers into bipolar filaments. NMHC phosphorylation or binding of the calcium-binding protein Mts1 promotes NM-2 filament disassembly. NMHC phosphorylation impairs Mts1 binding. Mechanisms underlying NMHC dephosphorylation are unknown

NM-2 motor activity, activation, and assembly state are determined by the reversible phosphorylation of both the NMHC and the associated RLC (Fig. 3). Regulation of NM-2 activity differs between higher and lower eukaryotes. RLC phosphorylation increases the enzymatic activity of *Dd* myosin-2 in vitro, but is dispensable since an unphosphorylatable RLC mutant fully rescues the phenotype of *Dictyostelium* RLC null cells [32]. In worms, flies, and mammals, RLC phosphorylation activates the enzymatic activity of the NM-2 holoenzyme and triggers the assembly in higher order filaments and hence actomyosin-mediated contractility [33]. At the amino acid level, the highly conserved residues S19 and T18 of the RLC constitute the primary and secondary phosphorylation site, respectively. In vitro, mono-phosphorylation of S19 enhances myosin ATPase activity, motor activity, and filament assembly [34–37]. This is discussed in greater detail below. Simultaneous di-phosphorylation of T18 and S19 further enhances the actin-activated ATPase activity and filament assembly [38, 39]. The activation of NM-2 by phosphorylation of the associated RLC controls assembly and activation of the holoenzyme to produce force on F-actin and serves as an indicator for active NM-2 in cellular studies.

RLC kinases include Rho effector and myosin light chain kinases (MLCK) [33, 40–42]. Regulated by respective upstream signals, phospho-signaling cascades converge either in Ca^2+^/calmodulin- or Rho signaling. The former activates MLCK [33], while the latter activates downstream effectors of the Rho family of guanosine triphosphatases (GTPase). The small GTPases RhoA and Cdc42 mediate the activation of Rho kinase (ROCK) and related effector kinases such as citron kinase, as well as the myotonic dystrophy kinase-related Cdc-42-binding kinase (MRCK) [43–45]. In contrast to MLCK, all other RLC kinases identified so far are not RLC-specific and act on a subset of cellular targets.

Protein kinase C (PKC) phosphorylates residues S1, S2, and T9 of the RLC. Phosphorylation of these sites decreases both the actin-activated ATPase activity and the affinity of MLCK for the RLC, thereby preventing NM-2 activation [46, 47]. Whereas the inhibitory S1/S2 phosphorylation is associated with mitotic arrest and stress fiber disassembly in live cells, inhibitory triple-phosphorylation of the RLC is not regarded as an important regulatory mechanism [48, 49].

RLC dephosphorylation is exclusively accomplished by myosin light chain phosphatase (MLCP). MLCP itself is highly regulated by numerous signaling loops including RhoA-ROCK signaling. RhoA-ROCK activity enhances RLC phosphorylation, both by inhibiting MLCP activity through the inhibitory phosphorylation of its regulatory myosin-binding subunit (MYPT) and direct RLC phosphorylation [50]. In agreement with the absence of RLC-phosphorylation as an important regulatory mechanism in *Dictyostelium*, no apparent orthologs of RhoA, ROCK, or MYPT have been identified [33].

Several reports implicate NMHC phosphorylation to regulate filament dynamics. Isoform-specific phosphorylation sites span the coiled-coil region and the NHT [51–53]. In vitro, kinases involved in NMHC phosphorylation include PKC, casein kinase 2 (CK2), and the ion channel kinases TRPM6 and TRPM7 [52, 54, 55]. Specific NMHC phosphatases have not yet been identified.

Studies with recombinant NM-2A and -2B tail domains suggest that NMHC phosphorylation inhibits filament formation by shifting the monomer-filament equilibrium towards the monomeric pool [56]. CK2-dependent phosphorylation of S1943 of NM-2A inhibits binding of Mts1 (also known as S100A4), a metastasis-associated protein. Mts1 promotes the disassembly of NM-2 filaments in an isoform-specific manner by sequestration of NM-2A in the disassembled state [56, 57]. In vitro, spectroscopic studies indicate that Mts1 promotes NM-2A rod fragments to disassemble from preformed hetero-assemblies of NM-2A and NM-2B [28].

Live cell studies on human carcinoma cells producing phosphomimetic NM-2A mutants S1943E and S1943D reveal increased migration rates, cell protrusions, and focal adhesions, when compared to wild-type NM-2A or the nonphosphorylatable NM-2A mutant S1943A [58]. Moreover, NMHC-2A phosphorylation during epithelial–mesenchymal transition (EMT) promotes enhanced motility and invasiveness of mesenchymal cells, possibly by a redistribution of NM-2 from posterior to anterior regions [59]. A phosphorylation-dependent turnover from distal to anterior regions of the lamellum has been reported for mutant NM-2A, either lacking the NHT or carrying the S1943A mutation [51]. NMHC phosphorylation hence prevents over-accumulation and mislocalization of NM-2 isoforms. The phosphorylation-dependent NM-2 turnover is required for its intracellular redistribution and the well-organized spatial and temporal controlled formation of local contractile actomyosin modules [51, 60–62]. Even though NM-2 filament formation in live cells is less well understood, NMHC phosphorylation appears to form a viable basis for the local fine-tuning of filament formation.

## Autoinhibition

Another regulatory mode of controlling NM-2 activity is mediated by the protein’s intrinsic ability to adopt an autoinhibitory conformation [63, 64]. This feature seems to be conserved among conventional myosins since it has also been described for smooth, cardiac, and skeletal muscle myosin [63, 65]. However, the molecular mechanism underlying the conversion of the inactive state to the active state remains to be resolved. Electron microscopic studies of unphosphorylated NM-2A homodimers reveal intramolecular head-to-head as well as head-to-tail interdomain interactions, bringing the two motor domains in close proximity [63, 65]. This conformation establishes contacts between the actin binding region of one head (blocked head) with the converter region of the second head (free head). This conformation impairs actin binding to the blocked head. In turn, contacts between the blocked head and the converter region of the free head inhibit the catalytic activity of the free head by blocking the nucleotide binding site. This double-negative feedback mechanism inactivates the enzymatic activity of both heads [63]. This structural model is supported by kinetic studies that demonstrate the autoinhibitory conformation to be enzymatically inactive, hence preventing constitutive activation [66, 67]. RLC-phosphorylation relieves the autoinhibited conformation, thereby promoting the adaption of a kinetically active and assembly-competent extended NM-2 conformation (Fig. 3). Sedimentation assays attribute faster sedimentation velocities to the compact conformer (10S) and slower sedimentation coefficients to the extended molecule (6S) [68]. The 10S conformer is proposed to constitute an assembly-competent NM-2 pool in equilibrium with NM-2 filaments [69]. The strictly regulated interconversion between the 10S conformer and filaments possibly reflects the spatiotemporal control of myosin-mediated contractility [69].

## Kinetic and mechanical properties

A detailed kinetic analysis of NM-2 isoforms can be performed with recombinant constructs typically produced in the baculovirus/Sf9 system. Constructs comprising the motor domain or fusions of the motor domain with an artificial lever arm are well suited for studying the kinetic properties. These myosin fragments are constitutively active and display the enzymatic properties of the phosphorylated holoenzyme [70]. Mechanical parameters like load dependence and processivity were determined using HMM fragments that require RLC phosphorylation for full activation [71, 72].

Comparative analysis of the kinetic parameters of fully activated mammalian NM-2 isoforms reveals subtle differences in the rate and equilibrium constants that determine the ATPase cycle (Table 1) [9–11]. These differences are responsible for the distinct enzymatic properties of NM-2 isoforms, reflecting their functional divergence and cellular roles. General features of NM-2 isoforms are a slow actin-activated ATP turnover and a low degree of coupling between the actin and nucleotide binding sites. Moreover, NM-2A and -2B show apparent second-order rate binding constants for ATP that are much smaller in the presence of F-actin [9–11]. An important functional property of NM-2 isoforms is that ADP binds to actin-bound NM-2 heads several times faster than does ATP [the ratio of the second-order ADP and ATP binding rate constants (**k**_**+AD**_ **/K**_**1**_ **k**_**+2**_) is approximately 20 in NM-2A, 10 in NM-2B, and 2.5 in NM-2C]. This feature provides a basis for efficient substrate inhibition by ADP, thus modulating the duty ratio and sliding velocity of NM-2 isoforms. NM-2 isoforms are amongst the slowest myosins characterized, in terms of the velocity at which they translocate actin filaments in the in vitro motility assay. NM-2A propels actin filaments 2–3 times faster than NM-2C or -2B [11, 73, 74]. The duty ratio is isoform-dependent: whereas NM-2A shows a low duty ratio similar to smooth and skeletal muscle myosins, NM-2B and NM-2C show slightly higher duty ratios [9–11]. Additionally, at least the duty ratio of NM-2B and NM-2C can be modulated by physiological changes in the concentration of ADP and free Mg^2+^ ions [9, 11]. Biochemical studies suggest that NM-2A and -2B dimers can bind to adjacent actin monomers in a two-headed conformation, a property typically associated with high duty ratio myosins [9, 72, 75, 76].

**Table 1:** Kinetic constants and mechanical properties of human noninserted NM-2 constructs

Parameter	NM-2A [10, 73]	NM-2B [9]	NM-2C [11]
Steady-state ATPase activity			
Basal (s^−1^)	0.013 ± 0.004	0.007 ± 0.001	0.06 ± 0.01
k_cat_ (s^−1^)	0.17 ± 0.005	0.13 ± 0.01	>0.23^a^
K_ATPase_ (μM)	72 ± 4	59 ± 3	>140
ATP binding to myosin and actomyosin			
K_1_k_+2_ (μM^−1^s^−1^)	1.03 ± 0.14	0.65 ± 0.06	0.37 ± 0.01
**K**_**1**_ **k**_**+2**_ (μM^−1^s^−1^)	0.14 ± 0.003	0.24 ± 0.02	1.02 ± 0.01
ADP binding to myosin and actomyosin			
k_+D_ (μM^−1^s^−1^)	0.55 ± 0.06	0.81 ± 0.23	0.54 ± 0.02
**k**_**+AD**_ (μM^−1^s^−1^)	2.72 ± 0.16	2.41 ± 0.13	2.56 ± 0.06
**k**_**+AD**_ **/K**_**1**_ **k**_**+2**_	≈20	≈10	≈2.5
ADP release from myosin and actomyosin			
k_−D_ (s^−1^)	1.12 ± 0.13	0.48 ± 0.11	0.42 ± 0.01
**k**_−**AD**_ (s^−1^)	1.72 ± 0.38	0.35 ± 0.03	0.78 ± 0.01
ADP affinity			
K_D_ (μM)	1.5 ± 0.4	0.65 ± 0.3	0.85 ± 0.12
**K**_**AD**_ (μM)	0.8 ± 0.2	0.15 ± 0.03	0.19 ± 0.07
F-actin affinity			
**K**_**A**_ (nM)	<10	<3	≈4.5
**K**_**DA**_ (nM)	≈20	<1	≈19
Duty ratio^b^	≈0.29	≈0.1	≈0.34
in vitro sliding velocity (nm s^−1^)	≈300^c^	<100^d^	≈90^c^

Kinetic parameters in the presence of F-actin are highlighted in *bold*. Subscripts *A* and *D* refer F-actin and ADP, respectively

^a^The value given at 140 μM F-actin

^b^Calculated from k_cat_/**k**_**−AD**_

^c^Temperature = 30 °C

^d^Temperature = 25 °C, *Gg* NM-2B HMM [74]

Three-bead optical trap assays show processive stepping along F-actin for a chicken NM-B HMM construct, supporting a model in which NMIIB can readily move in both directions at stall, which may be important for a general regulator of cytoskeleton tension [71, 75]. As suggested by Norstrom et al. [71], this property may provide a mechanism for disassembly of fascin-actin bundles. NM-2 activity can fluidize actin networks under conditions promoting filament sliding or by directly inducing disassembly of F-actin bundles [60]. The NM-2 mediated disassembly of actin bundles is a two-step process consisting of the unbundling of F-actin bundles into individual filaments and depolymerization of the latter [77].

All NM-2 isoforms assemble into bipolar structures that are considerably shorter than those formed from skeletal muscle myosin. In the context of NM-2 bipolar filaments, the effective duty ratio of the array is high enough to allow the continuous interaction with neighboring actin filaments, allowing processive movement along F-actin. Load further regulates and coordinates the interaction between F-actin and NM-2 isoforms [72]. Especially NM-2B shows a pronounced load-dependent product release. Under forward load, NM-2B accelerates the cycle of interaction with F-actin. Resistive load increases the duty ratio. Hence, NM-2B behaves like a cross-linker and prolongs tension generation on F-actin [72].

As reviewed by Lecuit et al. [60], cross-linked actin networks can stiffen when strained by internal or external forces. Rheologic studies suggest that NM-2B efficiently promotes cross-linking of F-actin in viscoelastic networks that display stress stiffening. Creep tests show that acto·NM-2B·ADP networks undergo viscous deformation and shear thicken at high stresses [78]. In contrast, short-lived interactions between NM-2B and F-actin in the presence of ATP restrict efficient cross-linking [78].

The motor function of all NM-2 isoforms can be selectively inhibited by the small molecule inhibitor blebbistatin in vivo and in vitro [79–81]. Structural studies using the *Dd* myosin-2 motor domain show blebbistatin to bind at the apex of the large cleft that divides the 50-kDa domain and close to the ATP binding pocket [82]. Kinetic studies indicate that blebbistatin binds with a high affinity to the myosin·ADP·P_i_ complex. The resulting slow phosphate release step inhibits formation of strong actin-binding states [79, 80]. As uncompetitive inhibitor, blebbistatin neither interferes with nucleotide binding nor the interaction with F-actin [79]. In the absence of ATP and the presence of ADP, blebbistatin appears to stabilize a strong actin-binding pre-powerstroke myosin intermediate [83].

Phalloidin binding to F-actin was shown to perturb the interaction of some NM-2 isoforms with F-actin. Phalloidin and fluorescently labeled phalloidin conjugates are commonly used in imaging and in vitro applications to visualize F-actin and to investigate actomyosin interactions. Kinetic and functional studies indicate that phalloidin perturbs the interaction of NM-2A and -2C with F-actin, whereas the interaction between NM-2B and F-actin appears less affected [84]. Therefore, isoform-specific interactions between actin filaments formed from α, β or γ actin and NM-2 isoforms are best studied in the absence of phalloidin and its conjugates.

## Development

NM-2 isoforms display a vast tissue distribution and most cells express a set of isoforms, but no consistent expression and intramolecular localization pattern has emerged [5, 31, 85]. Certain cell types predominantly or exclusively express one particular NM-2 isoform. For example, platelets and spleen produce exclusively NM-2A [85, 86], whereas neuronal tissues such as the cerebellum and the spinal cord are enriched in NM-2B [86, 87].

Their functional divergence allows NM-2 isoforms to assume distinct roles at specific developmental stages, as seen in mouse models. Germ line ablation of NMHC-2A causes embryonic death by day E6.5 due to defects in cell–cell adhesion, visceral endoderm formation, failure to organize normal germ layers, and the resulting impairment of the embryo to undergo gastrulation [12, 88]. Caused by cardiac and brain defects, NMHC-2B ablation results in embryonic lethality between day E14.5 and birth [89, 90]. Possibly due to the delayed *MYH14* expression in mouse embryonic development starting at day 10.5, NMHC-2C knockout mice show no obvious phenotype and survive to adulthood [86]. Mass spectroscopic analysis of adult mouse tissues reveals that overall most tissues produce significantly less NM-2C than NM-2A and -2B [86]. In contrast, NM-2C forms 15–45 % of the total NM-2 pool in transformed cells and cell lines such as the monkey kidney fibroblast cell line COS-7 and the colon adenocarcinoma cell line HT29 [86]. The simultaneous production of relatively low amounts of NM-2C along with high amounts of NM-2A and NM-2B in murine tissue and organs, such as the adult cerebellum, the cerebral cortex, the spinal cord, and kidneys, raises the question to what extent NM-2 isoforms can functionally replace each other [86]. Three factors—the total NM-2 content in the tissue, their motor activity, and scaffolding properties—appear to determine the extent to which one isoform can substitute for another [12]. Comparison of various phenotypes of genetically modified mice models suggests that isoform-specific enzymatic properties are less susceptible to substitution than tail domain-mediated scaffolding properties, indicating the capacity for partial compensation [12].

NM-2B and NM-2C exist in the form of several splice variants. All three NM-2B splice forms are produced in adult mouse brain in a spatially restricted manner [91]. The importance of their spatial and temporal splice heterogeneity is reflected by the neuron-specific expression of NM-2B1 and -2B2 during rodent brain development [19]. NM-2B1 ablation causes the abnormal migration of facial neurons and is associated with the development of hydrocephalus during mouse embryogenesis [91]. NM-2B mRNA is predominantly detected in various regions of the embryonic and neonatal brain, whereas the NM-2B2 mRNA level is low. Postnatal up-regulation of NM-2B2 mRNA is observed during dendritogenesis and synaptogenesis in cerebellar Purkinje cells [19, 92]. NM-2B2 ablation results in abnormal maturation of Purkinje cells in the developing mouse cerebellum, as manifested by a motor impaired phenotype [91]. The available kinetic and mechanic data for baculovirus-expressed constructs of NM-2B1 indicate enhanced actin-activated steady-state ATPase activity and in vitro translocation of actin filaments, when compared to the noninserted splice form NM-2B. Both NM-2B and NM-2B1 are regulated by RLC phosphorylation [74]. In contrast, NM-2B2 appears to lack actin-activated ATPase activity, motor activity, and regulation by RLC phosphorylation [93].

Both the noninserted and the C1 inserted splice variants of NM-2C are ubiquitous in their tissue distribution, whereas the expression of the C2 inserted gene product is confined to neuronal tissues [5, 94]. The unbalanced splicing of NMHC-2C with the prevalent production of the noninserted NM-2C splice form in human myostonic dystrophy type (DM1) muscle, in combination with the down-regulation of both the *MYH14* transcript and protein levels, promotes the development of DM1 histopathological features [95]. NM-2C1 is the only splice variant found in tumor cell lines [94]. Comparisons of numerous human tumor and nontumor cell lines, which were derived from the same tissue, indicate increased levels of NM-2C1 production in tumor cells [18, 94]. In the human A549 lung tumor cell line, small interfering RNA (siRNA) silencing of NM-2C1 delays cell proliferation by interfering with a late step in cytokinesis [94]. Reintroduction of NM-2C1 can rescue the phenotype. The noninserted splice form NM-2C can partially compensate the decreased proliferation rate, while NM-2A or -2B overproduction is ineffective [94].

In contrast to the equivalent NM-2B splice variant, recombinant HMM constructs of NM-2C2 with an expanded loop-2 are constitutively active and do not require RLC phosphorylation [18].

## Cell adhesion and morphogenesis

Morphogenesis involves the translation of biochemical signaling pathways into forces that move cells. NM-2-mediated contraction and adhesive forces control embryonic epithelial morphogenesis and organogenesis. Moreover, NM-2 motor activity is at least in part responsible for the cytoskeletal reorganization during epithelial morphogenesis that determines cell intercalation, invagination, shape, and rotation [96].

Gastrulation in *Drosophila* encompasses active cell shape changes that lead to the formation of ectoderm, endoderm, and mesoderm layers. Gastrulation is followed by germ-band extension, which leads to an anterior–posterior axis elongation of the epithelial layer that forms the thorax and the abdomen of the embryo. All stages of gastrulation in *Drosophila* require the polarized distribution of NM-2 and adhesion remodeling [96]. Before gastrulation, the embryo forms a single layer of cells arranged in a cylindrical shape and NM-2 localizes to the inner surface. At the beginning of gastrulation, RhoA signaling leads to an accumulation of NM-2 to the apical sites of the constricting cells, and actomyosin-mediated compression pushes the inner portion of the cells inwards, thereby creating a furrow that invaginates [97]. During dorsal closure of the epithelium, a late event in gastrulation, NM-2 localizes to the leading edge where it creates a tension force that pulls adjacent cells together as it contracts. Studies by Franke et al. [97, 98] suggest that NM-2, in either the leading edge cells or the underlying layer, is sufficient for dorsal closure. Germ-band extension in *Drosophila* is realized by the NM-2-driven disassembly of adherens junctions and planar junction remodeling, processes required for cell intercalation and hence anterior–posterior axis elongation [99]. Deletion of *zipper*, the gene encoding the *Drosophila* NM-2 heavy chain, is lethal because of failure in dorsal closure [97]. In analogy to the function of *zipper* in the model organism *Drosophila*, NM-2A knockout mice die because of defects in the visceral endoderm development and the failure of the embryo to undergo gastrulation [1]. NM-2B ablation causes specific defects in cardiac and brain organogenesis [90, 91].

Neural tube formation in vertebrates and *Xenopus* depends on cell shape changes via the apical positioning of actomyosin in neurepithelial cells [100, 101]. The actin binding protein Shroom3 localizes to the apical tip of adherens junctions and the apical junction complex (AJC) and directs the spatial recruitment of ROCK as well as the assembly of an actomyosin network associated with the AJC. ROCK-induced actomyosin contractility further mediates the Shroom3-induced apical constriction [100, 101]. Interestingly, ROCK is activated by the small G-protein Rap1 and not RhoA, which suggests the Rho-ROCK complex and Shroom3 work in separate pathways that converge to mediate constriction [100, 101]. Furthermore, Shroom3 is a regulator of the microtubule cytoskeleton, suggesting that the coordinated activity of the actin and the microtubule cytoskeleton are essential during epithelial morphogenesis in the developing vertebrate [102].

The lineage commitment of mesenchymal stem cells (MSC) and precursor cells is controlled by Rho-ROCK signaling and NM-2 activity. Regulating factors are extracellular matrix (ECM) stiffness and cellular confluence [97]. The mechanical properties of the ECM significantly determine cell fate: soft matrices are neurogenic, stiffer matrices are myogenic, and rigid matrixes are osteogenic [103]. Stiff substrates promote focal adhesion growth and elongation, and actin assembly follows the trends in adhesion assembly [103]. NM-2 directly promotes the assembly of focal adhesion and senses cortical actin structures linked to focal adhesions, thereby providing force transmission from the cell to the ECM [104, 105]. Therefore, prominent adhesions of stiff substrates are correlated to increased cytoskeletal tension through actomyosin-mediated contractility, which generates high tension forces that pull on the surface and promote differentiation towards the osteoblast lineage [97, 103]. Overexpression of either Rho or ROCK stimulates actomyosin contractility and supports differentiation to osteoblasts [97]. Chemical inhibition of NM-2 or MLCK blocks all elasticity-directed lineage specification on any substrate [103]. NM-2 exerts force through focal adhesions in mechanisms of matrix sensing, hence contributing to elasticity-driven lineage specification [103].

Cellular confluence promotes the commitment of precursor cells: sparse MSC densities promote the commitment towards osteoblasts, whereas confluent MSC differentiate to adipocytes [106]. Inhibition of actomyosin filament formation triggers preconfluent human MCS to adipogenesis instead of osteogenesis [105]. Single MSC plated on small substrate areas show a round morphology and undergo adipocytic differentiation. On large substrate areas, cells retain an elongated shape that triggers osteogenesis [105, 106].

Cell shape changes are linked to Rho-ROCK signaling and hence the commitment of MSC. Inactive Rho-GDP is the predominant Rho species in confluent or rounded MSC and promotes adipogenesis and chondrogenesis [106]. A round cell shape decreases the area with a rigid surface and prevents the cell from generating actomyosin-mediated tension and contractility [97]. Consistently, constitutively active Rho inhibits adipocyte differentiation. Overexpression of Rac, which opposes the actions of Rho-ROCK signaling, inhibits cytoskeletal contraction and promotes lineage commitment to adipocytes and chondrocytes [97, 106]. Active Rho-GTP in spread cells activates ROCK and filament formation. Actomyosin-mediated contractility inhibits adipogenesis and chondrogenesis and promotes osteogenesis [97, 106].

Besides the role in differentiation of MSC, NM-2 regulates the survival threshold of human and mouse embryonic stem cells (ES) [107]. ES show increased survival after treatment with Y-27632, an inhibitor of ROCK [108]. Genetic or pharmacological inhibition of NM-2 enhances the survival and self-renewal of pluripotent stem cells and is associated with an increased expression level of self-renewal regulators such as Nanog and Oct3/4 [107]. Similarly, enhanced survival is associated with murine ES lacking NM-2A [107]. In contrast, NM-2B-ablated ES show survival rates comparable to those of wild-type cells, indicating distinct functions of NM-2 isoforms in ES cell death [107]. NM-2 also regulates the cell–cell adhesion of human and mouse ES cells via a Rho-ROCK signaling pathway [109]. ROCK inhibition reveals that myosin-mediated cell–cell contacts are dispensable for maintaining the pluripotent function of ES [109]. In this context, the cell–cell contact-free growth of ES plated on E-cadherin-coated plates may account for the modulation of ROCK signaling, since both proteins mutually control cell adhesion [109].

In contrast, myosin-mediated cell–cell adhesions and tension generation of NM-2A on actin filaments, which are linked to the E-cadherin/beta-catenin complex, are required to maintain the adhesion complex in the developing mouse embryo [1]. Amongst other abnormalities, NM-2A-ablated embryos and ES demonstrate a loss in cell–cell adhesion in combination with a decrease in E-cadherin and β-catenin localization at cell–cell adhesion sites [1]. The defect in cell–cell adhesion causes cells to detach from the surface of embryoid bodies and to migrate out from the cell cluster, whereas wild-type embryoid bodies retain a cohesive morphology [1]. Embryonic lethality of NM-2A-ablated mice may be caused not only by cell–cell adhesion impairment but also by defects in ES differentiation, as outlined above [107]. Loss of cell–cell adhesion in NM-2B-ablated mice is the cause of hydrocephalus [110]. The absence of NM-2B in the apical border of the cells lining the spinal canal enable the underlying neuroepithelial cells to invade the canal, thereby interrupting the cerebral spinal fluid flow [110].

## Cell Migration

Directed cell migration is an essential process in the development and maintenance of multicellular organisms and is associated with cellular functions such as immunity, wound and tissue repair, angiogenesis, and normal and cancerous motility. Cell migration requires front-back polarization, membrane protrusion, adhesion formation and disassembly, cell body translocation, and rear retraction and is associated with dynamic interactions between NM-2, F-actin, the microtubule network, and focal adhesions [111, 112]. The coordinated adhesion assembly at the front and disassembly at the rear between the cell and a substrate is a prerequisite for cell migration [42]. ECM-cell adhesions are force-sensing integrin-based assemblies that provide a mechanical link between the actomyosin cytoskeleton and the ECM. The formation of nascent focal adhesions is NM-2 independent, whereas the formation, growth, and maintenance of mature focal adhesions require NM-2 motor activity and actomyosin contractility [42, 113]. During all stages of the migratory process, NM-2 isoforms orchestrate the dynamic spatial and temporal reorganization of the actin and to a lesser extent microtubule cytoskeleton [114]. This requires a capacity for local restricted self-organization, including the simultaneous performance of discrete sets of tasks in response to external trigger events during interphase and a distinct precisely timed and highly synchronized set of functions during mitosis. At the heart of the ability to perform these independent tasks within the cytosolic compartment is the occurrence of two cytosolic actin isoforms, namely β-*cys*-actin and γ-*cys*-actin. Post-translational modifications and the interaction with actin binding proteins can amplify the diversity of the cytosolic actin isoforms. Tropomyosin (Tm) isoforms were recognized to be of particular importance for the spatial and temporal dynamics of NM-2-actin interactions in nonmuscle cells. Moreover, it has been demonstrated that the activity of myosin motor domains are differentially regulated by the Tm isoform composition of actin filaments. Thus, elevated production of Tm5NM1 in neuroepithelial cells was shown to promote stress fiber formation, cell spreading, and decreased motility. Increased TmBr3 levels induce lamellipodial formation, faster motility, and a reduction in the formation of stress fibers. Incorporation of Tm5NM1 into stress fibers specifically recruits NM-2A into these structures, while NM-2B becomes enriched at the cell periphery [115].

**Fig. 4:**
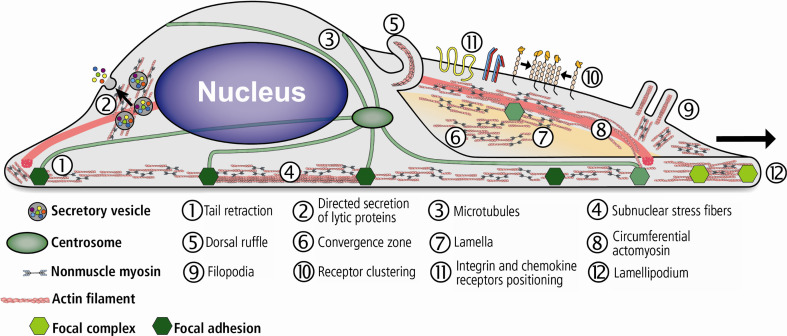
Schematic illustration of NM-2 cellular functions in cell migration, secretion and receptor positioning. During directed cell migration, actin polymerization and reorganization establish a protrusive leading edge at the anterior part of the cell. Cell protrusions are maintained by the contractile actomyosin network and stabilized by the formation of adhesive complexes (nascent focal adhesions) with the substratum. NM-2B localizes mainly to the lamellum, where it drives the retrograde flow. Actomyosin-mediated contractility posterior to the leading edge drive cell body retraction and translocation. Both processes coincide with the formation, growth, and maintenance of mature adhesion complexes (mature focal adhesions), which are NM-2-dependent. At the retracting rear, NM-2 is predominantly organized into stress fibers that promote local contractility. Adhesion complex disassembly causes detachment of the trailing edge from the substratum and enables cell displacement. NM-2 further mediates the cross-talk between the F-actin and the microtubule network. Actomyosin interactions further drive various processes such as vesicle transport, receptor-stimulated exocytosis of secretory vesicles, and receptor positioning

Migrating cells form actin-based cytoskeletal extensions consisting of distinct substructures, designated lamellipodium and lamellum. Both substructures differ in dynamic properties and protein composition. The lamellipodium contains a dense dendritic actin network and dynamic focal contacts. The polymerization of actin filaments with their plus ends oriented towards the plasma membrane is balanced by a myosin-powered, rearward movement of the lamellum actin meshwork known as retrograde flow. The lamellum is less dynamic than the lamellipodium and is characterized by linear actin bundles and mature adhesion sites [116, 117]. In general, NM-2 promotes F-actin anterograde flow in the cell body and retrograde flow in the lamellum [118, 119]. Behind the lamellum, which typically spans a broad area, actin bundles and meshwork move towards the cell front to create a ‘convergence zone’, where retrograde and anterograde actin motions merge and NM-2 is concentrated (Fig. 4).

Isoform-specific roles become prominently evident in processes such as the lamellar spreading of MDA-MB-231 breast cancer cells on an extracellular matrix, where NM-2 is recruited to the lamellar margin in a phosphorylation-dependent manner. Pharmacologic inhibition of either NM-2 or MLCK is associated with decreased migratory speed. SiRNA depletion of NM-2A impairs cell migration but enhances lamellar spreading. Depletion of NM-2B decreases both lamellar spreading and cell migration, highlighting the importance of both isoforms during cell migration and the preferential role of NM-2B in lamellar protrusions [120]. Fibroblasts from NM-2B-ablated mice display unstable and disorganized protrusions, but migrate with increased speed and decreased persistence, suggesting that NM-2B directs cell movement by coordinating protrusive activities and stabilizing cell polarity [121].

Rho GTPases play a major role in regulating NM-2 activity during cell migration. The activity of the associated Rho-dependent kinases that phosphorylate the RLC and thereby activate the NM-2 holoenzyme is under tight temporal and spatial control. RhoA is predominantly implicated in cell body and rear retraction via a ROCK-dependent mechanism. RhoA-ROCK signaling regulates the establishment of focal contacts and the organization of stress fibers by activating NM-2 activity [122]. Cdc42-dependent activation of MRCK is associated with lamellar actomyosin contractility, whereas stimulation of the Rac-MLCK pathway results in lamellipodial contractility [123]. The spatial segregation of the interplay between RLC-kinases and NM-2 isoforms hence causes symmetry breaking, leads to front-back polarization, and triggers directed migration of the cell [124, 125].

Isoform-specific functions of NM-2A and -2B are described in migrating cells, whereas less information is currently available on the role of NM-2C in active cell migration. NM-2A is predominantly found in the anterior region and in protrusions, but not at the leading edge. NM-2B localizes mainly to the center and the rear of migrating cells and is excluded from protrusions [85, 125].

During cell migration, NM-2 activation and the assembly of stress fibers are required for tension exertion on focal adhesions (Fig. 4). At the protrusive front of the cell, NM-2A supports dynamic focal contact turnover [125]. At the lamellum, NM-2-mediated contractility and substrate adhesion contribute to cell migration [119]. Moreover, recent data suggest NM-2A to directly mediate the actomyosin–microtubule cross-talk during cell migration. SiRNA-mediated depletion of NM-2A stabilizes microtubules near the leading edge and reduces the number of focal adhesions and stress fibers. As a consequence, cell contractility is reduced but cell migration increased. NM-2A-deficient cells further show increased membrane ruffling and an unusual expansion of microtubules into the lamellum. Both effects can be explained by the increased stability of microtubules, which drive membrane ruffling mainly by exerting force against the membrane in the absence of NM-2A. This suggests a function of NM-2A in mediating the cross-talk between the actomyosin and the microtubule system [112].

At the retracting cell rear, NM-2 is mainly organized into stress fibers and is involved in symmetry breaking and actin reorganization [122, 125]. NM-2A initiates the formation of actomyosin proto-bundles. NM-2B incorporated into these bundles stabilizes and enlarges them, thereby promoting the formation of extended rears [126].

NM-2B forms large and stable adhesion and actomyosin bundles that locally inhibit protrusions and adhesion turnover [125]. NM-2B might not be unique in its ability to form and stabilize a contractile rear because migrating B16 melanoma cells, which naturally produce NM-2C but not NM-2B, show normal front-back polarization, suggesting that the isoforms play analogous roles in creating a cell back [125].

The interplay between focal adhesion and actomyosin dynamics result in a specific balance between migration and adhesion, which determines the migration velocity. The size and density of adhesions decrease when NM-2 activity is inhibited by blebbistatin and increase upon MLCP inhibition. Increased NM-2 activity is associated with increased migratory speed [127]. In addition, early adhesion-site formation has the same periodicity as myosin-dependent edge retractions, suggesting a mechanical relationship between edge retractions and early adhesion-site formation [128, 129].

Experiments on the dynamics of adhesions-associated NM-2A and -2B filaments indicate isoform-specific functions during adhesion formation, maturation, and turnover. Actomyosin bundles containing solely NM-2A mediate initial adhesion maturation. The associated adhesion sites turn over in parallel with the actomyosin bundle. Incorporation of NM-2B into the bundles enlarges and stabilizes adhesions and abolishes dynamic adhesion turnover [126]. In contrast, NM-2A containing bundles at anterior parts of protrusions disassemble as the protrusions evolve. Incorporation of NM-2B in these bundles stabilizes them, possibly reflecting the higher actin affinity and duty ratio of NM-2B compared to NM-2A [126].

## Cytokinesis

Cytokinesis begins shortly after the onset of sister chromatid separation during the anaphase of mitosis. It comprises the final events in the cell cycle with the positioning and constriction of a contractile ring, followed by abscission, and the cutting of the midbody channel that forms the final bridge between the dividing daughter cells. An important role for NM-2 isoforms in these events is suggested by a large number of results. Deletion of *mhc*A in *Dictyostelium* abolishes cytokinesis [14]. Microinjections of pan-NM-2 antibodies or siRNA-mediated knockdowns prevent furrow ingression [130, 131]. NM-2B depletion in mice leads to defects in myocyte cytokinesis [90]. Specific NM-2 isoforms play an essential role during several morphological stages of cytokinesis, with their action again critically depending on the spatiotemporal regulation of RLC phosphorylation in higher eukaryotic cells [20].

The contractile ring that is formed during cytokinesis is composed of specific NM-2 and actin isoforms as well as associated regulatory and scaffolding proteins. These proteins assemble in the equatorial cortex after the selection and positioning of the cleavage plane by the microtubule cytoskeleton [132]. The contractile ring forms perpendicular and equatorially at the cell cortex to the anaphase spindle. During interphase, actomyosin-containing stress fibers disassemble and relocalize to the nascent cleavage furrow, where they provide the force that drives furrow ingression and constriction [133, 134]. The contractile ring is a highly dynamic structure that assembles and disassembles during each cell cycle. Cortical flow has been implicated in the transport of NM-2, F-actin, and other proteins to the contractile ring during its formation, with NM-2 motor activity also providing the force that drives the flow [135–137]. As suggested by Levayer et al. [138], actomyosin accumulation at the equator is promoted by two synergetic mechanisms that result in a centripetal actomyosin flow: the central spindle-dependent NM-2 activation (RhoA-dependent) promotes the recruitment of NM-2 at the equator, whereas astral microtubules inhibit (Rho-dependent) NM-2 recruitment to the cell periphery.

In some cells, NM-2 localizes to the ring prior to F-actin, suggesting that NM-2 directly contributes to actin assembly. However, NM-2 enzymatic activity is not required for the recruitment of either myosin or F-actin to the contractile ring [81, 139, 140]. Chemical inhibition of NM-2 motor activity inhibits cytokinesis but does not interfere with the equatorial localization of either actin or myosin, even though actin turnover is reduced. However, actin itself is highly dynamic in the contractile ring and dissociates from the equator in control cells, whereas it accumulates in blebbistatin-treated cells, which has been attributed to the lack in myosin motor activity [141, 142]. These findings indicate that NM-2 enzymatic activity is not required for the assembly of the equatorial cortex, but is essential for actin retention and its dynamic turnover [139, 141, 142]. Similarly, the ability of NM-2 to translocate actin appears not to be required for constriction of the cleavage furrow. Rather, the role of NM-2 in vertebrate cell cytokinesis involves the generation of tension to resist expansion of the contractile ring by binding and cross-linking of actin filaments [143].

The phosphorylation status of the NM-2 associated RLC changes during the cell-cycle of higher eukaryotes: Upon mitotic exit, S19 of the RLC becomes rapidly phosphorylated [33, 144, 145]. Phospho-specific antibodies indicate that the cleavage furrow is enriched in RLC phosphorylated NM-2 holoenzymes [146]. Overexpression of a nonphosphorylatable RLC disrupts cytokinesis by producing an abnormal, distorted cleavage furrow, which leads to a failure to complete cytokinesis [147].

MLCK, MLCP, and the two RhoA-dependent kinases, ROCK and citron kinase, localize to the cleavage furrow and regulate NM-2 activity during cytokinesis [148–150]. RhoA activation and its accumulation at the contractile ring are indispensable for furrow formation and ingression [151]. Both RhoA activity and the position of the cleavage furrow are mediated by the central spindle, suggesting a link between microtubule organization and RhoA activation at the equator [60, 144]. RhoA activation at the cleavage furrow leads to the temporal recruitment of its effector kinases: ROCK is recruited during late anaphase and stays at the furrow during cytokinesis, suggesting that the RhoA-ROCK pathway plays a role in contractile ring formation and cleavage furrow constriction [152]. Citron kinase colocalizes to the cortex of the cleavage furrow during telophase and cytokinesis and is involved in the stabilization of NM-2 binding partners and abscission, as outlined below [140, 150]. Inhibition studies in *Drosophila* suggest that citron kinase is dispensable for initiation and constriction of the cleavage furrow [153, 154]. Activated MLCP accumulates at the cleavage furrow and indirectly enhances the amount of RLC phosphorylation [155]. MLCK is recruited during late anaphase and telophase and might be involved in both the assembly of the contractile ring and its constriction [148, 149].

Besides the regulation of NM-2 activity via the phosphorylation of its RLC, regulatory and scaffolding proteins interact with NM-2 at the cleavage furrow and appear to be involved in its recruitment. Filamentous SEPT2 directly binds NM-2, which links the former to F-actin [133]. This interaction is required for NM-2 activation in interphase and cytokinesis [133]. Disruption of the interaction is associated with cleavage furrow instability and decreased RLC phosphorylation. SEPT2-containing filaments possibly form a scaffold that brings NM-2 and its associated RLC kinases in close proximity, thereby ensuring maximum NM-2 activation during the final stages of cytokinesis [133]. Another regulator of cytokinesis, anillin, binds to the phosphorylated NM-2 holoenzyme, F-actin, and septins [156]. RhoA activity recruits anillin to the equatorial cortex early in cytokinesis, where it organizes the contractile ring [156]. As part of the contractile ring, anillin restricts NM-2 contractility to the cleavage furrow during late stages of cytokinesis [156]. NM-2 leaves the contractile ring late in cytokinesis and anillin persists at the contracted furrow, where it is required for abscission [153, 156].

NM-2C1 is implicated in abnormal cytokinesis in cancerous cells and localizes to the midbody, whereas NM-2A is distributed throughout the cell and concentrated at the two opposite poles of the dividing daughter cells during the late stages of cytokinesis [94].

## Vesicle transport, endocytosis, and exocytosis

Collective findings suggest NM-2A and -2B to be involved in intracellular membrane fission of Golgi-derived vesicles and their transport between different compartments [157–160]. NM-2A transiently localizes with membranes of the trans-Golgi network (TGN) during vesicle budding and is found on a specific subset of Golgi-derived vesicles [161–163]. Proteolytic cleavage experiments suggest that NM-2A binds via its tail domain to Golgi stacks. This interaction is abolished in the presence of NMHC phosphorylation by CK2 [164]. These findings indicate that NM-2A is tethered to the Golgi membrane via its tail, while its motor domain interacts with F-actin [164]. The directed movement along F-actin might therefore extend Golgi membrane tubules or transport vesicles away from the Golgi complex [164]. This model is supported by studies showing that NM-2 is involved in retrograde transport of vesicles from the Golgi complex to the ER [159]. Recent work by Miserey-Lenkei et al. [157] demonstrates NM-2A and -2B to trigger fission of Rab6 transport carriers from the Golgi complex by interacting with Rab6 and F-actin. The GTPase Rab6 directly binds to the coiled-coil of NM-2 in a GTP-dependent manner, thereby recruiting NM-2 isoforms to the Golgi membrane [157]. Pharmacological or genetic depletion of NM-2 or actin polymerization is associated with a phenotype that produces long tubule stalks that radiate from the Golgi complex and fail to undergo fission [157]. NM-2A- and -2B and Rab6 localize to fission sites of these tubular precursors. Actin is recruited to the assembly sites where it is required for the detachment of Rab-6 positive transport cargoes from the stalks [157, 165]. Inhibition of either NM-2 or Rab6 impairs both the fission of Rab6 cargos from Golgi membranes as well as the trafficking of anterograde and retrograde cargo from the Golgi [157].

Recent work by Tang et al. [166] demonstrates NM-2A to participate in the formation of autophagosomes, organelles that capture cellular components and deliver them to the lysosomes for degradation. Agt1 kinase plays a crucial role in the induction of autophagosome formation. In *Drosophila*, overexpression of Agt1 results in aberrant cell morphology and triggers the reorganization of the actin cytoskeleton, mediated by activation of *zipper* [166, 167]. The Agt1/Ulk1 signaling pathway activates the kinases sqa/ZIPK in *Drosophila* and humans, respectively, which phosphorylate the *zipper* or NM-2A-associated RLC during starvation-induced autophagy. Consequently, knockdowns of either ZIPK or NM-2A lead to a decrease in the size and number of autophagosomes [166]. Activated NM-2 controls autophagosome formation by interacting with the transmembrane protein Atg9, as well as trafficking of Atg9-containing membranes from the TGN to the sites of autophagosome nucleation [166, 168]. This leads to the speculation that *zipper*/NM-2A may either act as a molecular motor that actively shuttles Atg9-containing vesicles between the TGN and the forming autophagosome or forms a complex with Rab6 at the TGN that promotes the fission of Atg9-containing vesicles [166].

During exocytosis, secretory vesicles derived from the ER or the Golgi network fuse with the plasma membrane and release their content into the extracellular space. Recent work has identified NM-2 isoforms to be involved at different stages of exocytosis. Confocal intravital microscopy on submandibular salivary glands of live rodents indicates that the β-adrenergic receptor-stimulated exocytosis of secretory vesicles is dependent on actomyosin activity [169]. Agonist-induced stimulation recruits both NM-2A and -2B onto the surface of fusing granules, where they function as part of the machinery that regulates the collapse of the granules after fusion with the apical plasma membrane [169]. In this context, the authors speculate that F-actin serves as a platform to recruit NM-2 to form a contractile scaffold that generates the force required for the collapse of the secretory granules [169]. Another example of NM-2-dependent exocytosis occurs in natural killer cells, which are lymphocytes of the innate immune system and important for defense against cancer and viral infection [170]. Natural killer cell cytotoxicity involves the formation of an immunological synapse between the natural killer cell and the target cell through which lytic granules are delivered to the target cells via exocytosis [171]. NM-2A inhibition or knockout blocks a step between the formation of mature synapses and lytic granule fusion with the cell membrane and promotes lytic granule exocytosis [170, 171].

Activation of the insulin receptor increases glucose transporter type 4 (GLUT4) vesicle exocytosis in adipocytes via a NM-2A-dependent mechanism. Insulin stimulates the MLCK-mediated RLC phosphorylation thereby triggering the translocation of NM-2A to the plasma membrane. There, the phosphorylated NM-2A holoenzyme exhibits a dual role in insulin-stimulated glucose uptake by facilitating GLUT4 vesicle fusion and regulating GLUT4 activity [172]. Insulin-stimulation does not change the localization of NM-2B, implicating that the two isoforms have different functions in adipocytes [172].

Collective findings implicate NM-2 isoforms to regulate the dynamic opening and closing as well as the size of the fusion pore during exocytosis [173]. Even though the exact role is not yet established, several studies have demonstrated NM-2 isoforms to control vesicle cargo-discharge kinetics by altering fusion pore conductance and gating in numerous cell types, including pancreatic β-cells (NM-2A-dependent), chromaffin cells, and neurons [174–176]. After the fusion of the vesicle with the membrane, actin polymerizes and coats the vesicle. Vesicle coating is independent from NM-2 activity and may reflect an early step in endocytotic recovery in some cells [177]. NM-2A directly affects post-fusion dynamics by regulating fusion pore opening and expansion in cells [173, 175, 177]. Chemical inhibition of either NM-2A or MLCK activity causes the closure of the fusion pore, indicating that NM-2A enzymatic activity is necessary to maintain fusion pore opening in pancreatic acinar cells [177]. Concomitantly, activated NM-2 slows down fusion pore closure upon cargo discharge during kiss-and-run exocytosis in neuroendocrine PC12 cells, possibly by modifying the subplasmalemmal actin cortex [178]. These findings suggest that NM-2 activity controls the amount of hormone released from vesicles in neuroendocrine cells by directly influencing the duration of fusion pore opening [178]. Different from full fusion exocytosis, NM-2 does not control the expansion of the fusion pore during kiss-and run exocytosis, where the fusion pore is resealed before complete dilation and cargo is not completely released [178].

Besides exocytosis, NM-2 participates in endocytosis and phagocytosis. Both processes describe the internalization of extracellular material by invagination of the plasma membrane to create an endocytic vesicle which enters the endosomal pathway.

The actin cytoskeleton is the key structure during receptor-mediated phagocytosis and involved in the formation and closure of the phagocytic cup [179]. Even though the role of NM-2 in this process has not yet been fully investigated, NM-2 mediated contractile activity is required during phagocytic cup assembly, squeezing and closure during the receptor-mediated ingestion [180, 181]. Several studies demonstrate the impact of cell type, receptor, and engulfed particle on downstream signaling pathways that recruit a special set of kinases, including Rho/Rac/Cdc42-dependent kinases to the nascent phagosomes where they regulate NM-2 activity as well as actin nucleation [179, 182].

Olazabal and coworkers demonstrated that the Rho-ROCK-phosphorylated NM-2A holoenzyme is required for F-actin recruitment to the phagocytic cup in complement receptor 3 (CR3)-, but not FcgR-mediated phagocytosis [181, 183]. During FcgR-mediated phagocytosis by macrophages, NM-2-mediated contractile activity promotes binding between the FcgR and ligands to facilitate the efficient extension and subsequent closure of phagocytic cups [180].

During retinal pigment epithelial phagocytosis of photoreceptor outer segments, the receptor tyrosine kinase Merkt is required for the spatial relocalization of NM-2A and -2B from the cell periphery to the phagosome [179]. Further, Merkt triggers the assembly and activation of the actomyosin complex at the ingestion that promotes the engulfment of photoreceptor outer segments [179]. NM-2 inhibition by blebbistatin or siRNA depletion of both NM-2A and -2B leads to a reduction in the number of ingested phagosomes, suggesting that both isoforms function in the phagocytic trafficking of photoreceptor outer segments [179].

Internalization of the chemokine receptor CXCR4 upon engagement by its agonists is facilitated by NM-2A in T-lymphocytes [184]. The agonist-mediated receptor endocytosis is inhibited by overexpression of NM-2A tail domain [184]. This study favors a model in which NM-2A serves as an adaptor protein that couples the membrane receptor to the endocytic machinery, thereby triggering the formation and uptake of CXCR4-bearing clathrin-coated endocytic vesicles [184]. NM-2A also mediates the cytokine interferon-gamma-induced endocytosis of tight junction proteins [185]. In this context, the RhoGEF-mediated spatial regulation of *zipper* has been shown to play a role in the initiation of E-cadherin endocytosis in *Drosophila* [186].

## Viral infection

Novel roles of NM-2 during several stages of viral infection emerge. As part of the viral entry machinery, NM-2 functions as herpes simplex virus type-1 (HSV-1) cellular entry receptor by directly associating with the viral envelope glycoprotein B (gB) on the surface of naturally permissive target cells [187]. The initiation of HSV-1 entry induces the cell-surface expression of NM-2 via a MLCK-dependent redistribution of cytosolic NM-2A [187]. Both antibody blockage and knockdown of NM-2A in permissive target cells inhibit HSV-1 infectivity whereas the overexpression of NM-2A in relatively HSV-1-resistant cell lines causes a high susceptibility to HSV-1 infection [187]. As a functional gB receptor, NM-2A mediates the broad HSV-1 infectivity by its ubiquitous expression in various human tissues and makes it a medicinally relevant drug target [187]. After HSV-1 entry into the host cells, viral nucleocapsids move to the nucleus and the viral genes are transcribed and translated. Late in infection, replicated DNA is packed in capsids. During viral egress, the capsids move from the nucleus to extracellular spaces [188]. Studies by Van Leeuwen et al. [189] implicate NM-2A to play a role in viral transport during herpes virus replication and viral egress. HSV-1 infection leads to the accumulation of cytoplasmic NM-2A in a perinuclear cluster, where it colocalizes with VP22, a major viral tegument protein. These perinuclear clusters are proposed to be possible viral assembly compartments where VP22 is incorporated into assembling virions. Pharmacological inhibition of NM-2A retards the perinuclear accumulation of VP22 clusters and the release of virus to the extracellular space with minor effect on the yield of cell-associated virus. These findings suggest a role of NM-2A during viral transport and egress [189]. This idea is supported by the observation that HSV-1 infection induces the formation of long plasma membrane protrusions that establish contacts with adjacent cells. NM-2 filaments run through the protrusions, and VP22-containing particles align and progress along these extensions to accumulate at the extremities of contact forming adjacent cells [189]. Similar, protrusions such as filopodia support the viral infection pathway of murine leukemia virus (MLV). Lehman et al. [190] have shown that an actin cytoskeleton and NM-2-mediated MLV surfing along filopodia towards viral entry sites at the cell body of permissive cells promotes MLV infectivity. Consequently, pharmacological inhibition of NM-2 disrupts viral surfing and reduces the viral infectivity [190].

Different from HSV1, bleb-associated macropinocytosis is the predominant mode of Kaposi sarcoma-associated herpes virus (KSHV) entry in its permissive target cells [191]. KSHV infection triggers the phosphorylation of C-Cbl. Phosphorylated C-Cbl associates with NM-2A and F-actin and is recruited to membrane blebs. The association with actomyosin leads to the C-Cbl-mediated ubiquitination of both NM-2A and actin. Actomyosin-mediated contractility possibly accelerates bleb retraction with the macropinosomes along with the viral particles. Concomitantly, blebbistatin treatment of the cells or shRNA knockout of C-Cbl causes defects in myosin-dependent blebbing and retraction during KSHV entry [191].

## Diseases

Mutations, alternative splicing, and misregulation of *MYH9* and *MYH14* and the associated changes in NM-2A and NM-2C are linked to the onset and progression of a number of serious human diseases. In contrast, disease-related *MYH10* mutations have not so far been characterized. Only indirect links exist between *MYH10* expression and disease processes, such as scar tissue formation following myocardial infarction, demyelination, and the inherited neurodegenerative disease, juvenile-onset neuronal ceroid lipofuscinosis (JNCL). JNCL is the most common form (1:12.500) of a genetically heterogeneous group of rare disorders known collectively as the neuronal ceroid lipofuscinosis (NCLs), or Batten disease. Classical JNCL, caused by CLN3 mutation, is a lysosomal storage disorder with onset between 4 and 8 years of age. The disease is characterized by accumulation of autofluorescent storage material and neurodegeneration. Symptoms include seizures, motor and cognitive regression, and progressive vision loss leading to complete blindness [192]. A direct functional and physical interaction between CLN3 and NM-2B has been linked to the role of CLN3 in mediating anterograde and retrograde trafficking [193]. The affected transport pathway connects the Golgi network, endosomes, autophagosomes, lysosomes, and the plasma membrane.

In neural tissue, the inhibition of NM-2B by blebbistatin or knock-down of *MYH10* by lentiviral shRNA promotes remyelination. The myelin-forming cells in the CNS are formed by differentiation of oligodendrocyte precursor cells (OPC) into myelinating mature oligodendrocytes (OL). Similar, after a demyelinating insult, remyelination involves OPC proliferation, their migration into the lesion, and differentiation into OL [194, 195]. Both migration and formation of myelin lamellae involve contributions from cytoskeletal motors [196, 197]. Recent results indicate that NM-2B critically contributes to these processes [198, 199].

After myocardial infarction, *MYH10* expression is upregulated in myofibroblasts during the early stages of cardiac remodeling. While invasion of the activated myofibroblasts into the damaged area is beneficial during early stages, their abundance has been linked to the formation of non-functional scar-tissue at later stages [200, 201]. The tight spatio-temporal control of NM-2B activity with the aid of specific inhibitors of myosin motor function [202, 203] therefore holds the promise that an over-shooting of the invasion of myofibroblasts can be prevented and an optimal ratio of myofibroblasts to myoblasts can be established [204].

A spectrum of autosomal-dominant disorders related to *MYH9* mutations are subsumed under the collective term *MYH9*-related diseases. As many as 40 different mutations have been mapped throughout the motor and the tail domain of NMHC-2A [205, 206]. Disease phenotype and genotypes are associated with congenital macrothrombocytopenia and the onset of clinically variable symptoms like deafness, progressive nephritis, and presenile cataracts [205]. A large case series of patients with *MYH9*-related diseases demonstrates that the site of *MYH9* mutation is a determinant of the clinical symptoms and features of the disease [205]. Patients carrying mutations within the catalytic NM-2A motor domain (R702C/H) develop severe thrombocytopenia, nephritis, and deafness before the age of 40 years [205]. Patients with mutations within the tail domain (e.g., D1424H/N/Y, E1841 K, R1933X, V1516 M) show no defects of clinical relevance [205, 207]. As demonstrated by Zhang and coworkers, studies of mouse models with *MYH9*-related diseases manifest similar phenotypes as observed in humans and hence serve as a good model system to study NM-2A and associated diseases [208].

Mutations in NM-2C are associated with hereditary deafness (DFNA4) [209]. Genome-wide linkage analysis identified an autosomal-dominant mutation which causes a complex phenotype associated with peripheral neuropathy, myopathy, hoarseness, and hearing loss [210]. Additionally, aberrant splicing of *MYH14* and the unbalanced expression of NM-2C splice-variants contribute to the molecular pathogenesis of DM1 [95].

Cancer metastasis and invasion require NM-2 mediated cell migration, contractility and cell adhesion dynamics [211]. NM-2 is directly and indirectly involved in cancer cell motility through overexpression and overactivation. Both mechanisms enhance cancerous cell migration, thereby contributing to tumor invasion and metastasis [212, 213]. NM-2A overexpression correlates with increasing numbers of metastatic lymph nodes, poor cancer differentiation, and advanced tumor stages of esophageal squamous cancer. SiRNA depletion of NM-2A in cancer cell line KYSE-510 results in increased cell–matrix adhesion, decreased cell motility, and reduced metastatic behavior [13].

In different carcinoma cell lines, ROCK overactivation causes enhanced RLC phosphorylation and direct activation of NM-2 driven cell migration [42, 214]. Cancerous breast cell migration and invasion is attenuated after either blebbistatin treatment or depletion of NMHC-2A and NMHC-2B [120, 214]. In contrast, non-cancerous mammary epithelial cell lines continue migration when treated with blebbistatin [112].

As well as overactivation through RLC phosphorylation, the direct interaction of NM-2A with the calcium-binding EF hand protein MtsI drives cancerous cell migration. Upregulation of Mts1 is associated with cancerous cell migration, metastatic progression, increased angiogenesis, and tumor invasion in numerous cell types. The direct interaction between Mts1 and NM-2A at the leading edge of migrating cells promotes directional motility, possibly by enhancing the NM-2A filament turnover. The associated increase in myosin-driven cell motility contributes to the increased metastatic capacity of cancer cells and highlights the importance of NM-2 isoforms during physiological homeostasis [215]. As reviewed by Helfman and coworkers, MtsI has been shown to interact with actin, nonmuscle tropomyosin, and p53, suggesting a role for MstI in cell motility and cytoskeletal rearrangements [216].

## Conclusion and outlook

The steadily expanding research on NM-2 has elucidated important functions of the family members in nearly all aspects of normal and aberrant cell physiology. Pivotal roles of NM-2 during development have been investigated and highlight the impact of NM-2 isoforms during embryogenesis and organogenesis. Tasks of NM-2 during cell migration and cytokinesis have been established that highlight the importance of the spatiotemporal regulation of the holoenzyme by kinases and phosphatases through major signaling pathways. In this context, NM-2 functions need to be addressed in a three-dimensional environment. New roles of the family members in endo- and exo-cytosis as well as other intracellular transport processes are emerging and will be the focus of future research. Despite our vast knowledge of NM-2 functions in general, many isoform-specific functions and interactions with partner proteins remain undefined. Questions that remain to be addressed include details of the interplay with the microtubule system, membrane interactions, and the cellular function of arguably the least-characterized family member NM-2C.
